# Changes in Bacterial Communities and Their Effects on Soil Carbon Storage in *Spartina alterniflora* Invasion Areas, Coastal Wetland Bare Flats, and *Sueada salsa* Areas

**DOI:** 10.3390/ijerph20054308

**Published:** 2023-02-28

**Authors:** Jiashuo Liu, Xiaoxiao Duan, Guo Li, Zhenjie Cai, Sijie Wei, Qixuan Song, Zheng Zheng

**Affiliations:** 1 Department of Environmental Science & Engineering, Fudan University, 2005 Songhu Road, Shanghai 200438, China; 2 School of Life Sciences, Nanjing University, No. 163 Xianlin Road, Nanjing 210023, China

**Keywords:** biological invasion, *Spartina alterniflora*, soil bacterial community, soil organic carbon

## Abstract

*Spartina alterniflora* is considered an invasive species that has affected the biogeochemical circle of carbon in coastal wetlands around the world. Nevertheless, it is still unclear how *S. alternation* invasion affects the carbon storage capacity of coastal wetlands as carbon pools through bacterial changes. Herein, bacterial communities and soil carbon content in coastal wetland native areas and *S. alterniflora* invasion areas were detected. It was found that an *S. alterniflora* invasion brought more organic carbon and resulted in the increase in *Proteobacteria* in bare flats and *Sueada salsa* areas. When decomposition capacity was not sufficient, large amounts of organic carbon may be stored in specific chemical forms, such as monosaccharides, carboxylic acids, alcohols, etc. The results have also shown that soil bacterial communities were highly similar between the bare flat and *S. alterniflora* invasion area, which is extremely conducive to the rapid growth of *S. alterniflora*. However, an *S. alterniflora* invasion would decrease total carbon contents and inorganic carbon contents in the *Sueada salsa* area. This is not conducive to the stability of the soil carbon pool and soil health. These findings may complement, to some extent, the shortcomings of the interaction between *S. alterniflora* and bacterial communities, and their joint effect on soil carbon storage.

## 1. Introduction

The coastal wetland flat is an ecological transition zone formed under the co-development of multi-water environments [1], which is located in the land-sea interaction zone. With unique hydrological conditions and physicochemical soil properties, it has been one of the most productive ecosystems on earth. Influenced by both ocean and land, the coastal wetland has an abundant environmental composition [2] that plays an important role in the global carbon cycle. Previous studies have indicated that the carbon storage of coastal wetland ecosystems, such as salt marshes, mangroves, and seagrass beds, accounts for more than 50% of all marine carbon storage [3,4]. However, coastal wetlands are also the zones most sensitive to climate change and most responsive to human activities. It is estimated that the carbon loss caused by the destruction of coastal wetlands per hectare is equivalent to 10–40 hectares of temperate forest [5]. Hence, the migration and transformation of soil carbon in coastal wetland carbon cycle research has attracted more attention.

Serving as the representative plant of coastal wetlands, *Spartina alterniflora* (*S. alterniflora*) was originally introduced for the purpose of protecting tidal flats and promoting sediment deposition [6,7]. Unfortunately, owing to its high adaptability and reproductive ability, *S. alterniflora* occupies the growth space of local plants, affects the material cycle and soil ecosystem of coastal wetlands [8], and threatens local biodiversity [9,10]. Current evidence preliminarily suggests that *S. alterniflora*’s influence on coastal wetlands is considered to be two-sided. Scholars paid more attention to the positive effects, and there have been significantly higher numbers of studies on the positive effects of *S. alterniflora* than those on the negative effects [11,12]. Research on the negative effects of *S. alterniflora* has increased recently, whereas these limited negative effects studies have merely focused on the different performance of *S. alterniflora* and other plants in the ecosystem [13,14,15]. In particular, the detailed interaction mechanism of *S. alterniflora* and soil microorganisms has not yet been clearly determined. For instance, Levin et al. and Ma et al. have focused on *S. alterniflora* changing the landscape and microtopography of coastal intertidal mudflats, as well as the living conditions of native species and the nutrient structure of ecosystems [14,15].

As an important biological type of coastal wetland ecosystem, soil bacteria plays a crucial role in the process of how changes to coastal wetlands are caused by an *S. alterniflora* invasion. Previous studies showed that the invasion of *S. alterniflora* could increase the richness of denitrification bacteria and denitrification ability in coastal wetland soil [16]. The gradient investigation of *S. alterniflora* was also carried out in the wetlands with different invasion conditions, and it was found that the increase in the saprotroph-symbiotroph was conducive to the colonization and expansion of *S. alterniflora* [17]. However, it is still unclear how an *S. alternation* invasion affects the carbon storage capacity of coastal wetlands as carbon pools through bacterial changes, which is also one of the most important ecological functions of coastal wetlands. Thus, it is necessary to compare the changes brought by an *S. alterniflora* invasion at the bacterial level and reveal how they interact with each other and what their joint effects are in the process of an *S. alterniflora* invasion.

In this paper, the intensity of soil carbon metabolism was substituted by the abundance of bacteria that control soil carbon metabolism. The bacterial community difference between *S. alterniflora* invasion areas and coastal wetland native ecology areas was defined by the difference in microflora phyla. At the same time, the changes of contents in the surface soil carbon storage, including soil organic and inorganic carbon, were detected. These changes in carbon contents were matched to changes in soil bacterial communities caused by an *S. alterniflora* invasion, in particular by combining changes in the bacteria that dominated carbon decomposition processes in coastal wetlands. Therefore, from the perspective of the interaction between plants and microorganisms, this paper reveals how *S. alterniflora* affects the soil bacterial community in coastal wetlands, and thus affects soil carbon storage in coastal wetlands. This contribution further provides a theoretical basis for the prevention and control of biological invasions and the protection of coastal wetlands’ soil health.

## 2. Material and Method

### 2.1. Study Area

**Figure 1:**
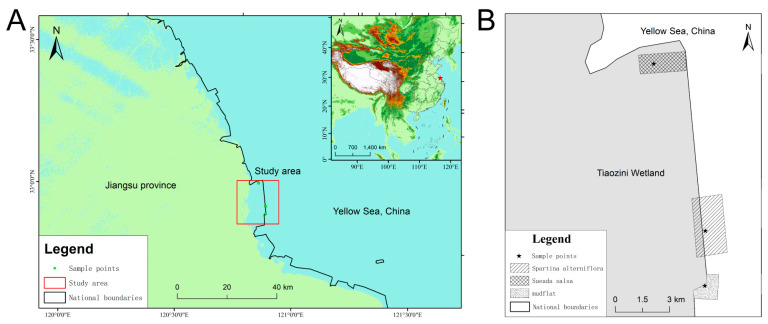
Study area diagram (**A**) and distribution diagram of sampling points (**B**).

The Tiaozini Wetland is a coastal wetland on the east coast of China, covering 600 km^2^ (Figure 1A). It is located in the transition area between the warm temperate zone and the subtropical zone (32°43′ N–32°52′ N, 120°53′ E–121°3′ E), with a mild climate and rich biodiversity. It is the central node of the migration route of East Asia-Australia migratory birds. In 2019, the Tiaozini Wetland was successfully selected as a protected World Natural Heritage Site. The texture of the wetland flat is mainly a silt flat, which has a strong ability to fix water and salt.

### 2.2. Samples Collection

Seventeen 5 m by 5 m quadrats were set up in three different ecological areas. Five different sample points were set up in each quadrat. Soil samples of 0–20 cm were collected from different sampling points in the same quadrat and mixed together to produce a sample. Finally, there were seventeen different soil samples collected at 0–20 cm in three different ecological areas. Eight soil samples (GT-1 to GT-8) were collected from a bare flat (GT). Four soil samples (JP-1 to JP-4) were collected from the *Sueada salsa* area (JP) that had been growing for more than ten years. These two areas were the native ecological areas of the coastal wetland. In addition, five samples were collected from an *S. alterniflora* invasion area (SA), including a perennial *S. alterniflora* growth area (SA-S-1,2,4), an *S. alterniflora* mature area (SA-S-P), and an *S. alterniflora* birth area (SA-2). All the sampling areas are located in the closed area of the World Natural Heritage protected area, with minimal human disturbance. Information on the samples and sampling locations are shown in Table 1.

### 2.3. Soil Carbon Content Detection

The carbon content in the sample was detected using a TOC-L SSM from the Shimadzu Company, which was able to directly detect the carbon content in soil solids and automatically acidified the soil to remove carbonates during the detection of inorganic carbon (IC). Each sample was freeze-dried, ground, and passed through 100 mesh screens. Each test was performed using 500 mg samples, and each sample was repeated three times. The data of soil total carbon content (TC) and inorganic carbon (IC) content was able to be obtained directly through instrument detection. The soil organic carbon content (SOC) of the sample is obtained from the difference between the TC and the IC of the sample.

### 2.4. DNA Extraction and PCR Amplification

DNA was extracted from wetland flat soil samples using the E.Z.N.A soil kit (Omega Bio-tek, Norcross, GA, USA). Three copies of each sample were extracted, and the resulting DNA was mixed. DNA concentration and purity were measured by NanoDrop2000, and quality of extracted DNA was measured using 1% agarose gel electrophoresis. The V3-V4 region of bacterial DNA was amplified via a polymerase chain reaction (PCR) using 338FmodF (5′-ACTCCTACGGGAGGCAGCAG-3′) and 806RmodR (5′-GGACTACHVGGGTWTCTAAT-3′) primers [18]. The two primers have increased the coverage of sequences available in the Ribosomal Database Project (RDP) and have been widely and successfully used in many previous studies for Illumina-based surveys of bacteria [19]. Detection results of PCR products from all samples were collected in a set for paired-end sequencing.

### 2.5. Illumina Miseq Sequencing

Sequencing was performed on an Illumina Miseq instrument from the Shanghai Personalbio Technology Co., Ltd. (Shanghai, China). After obtaining the sequencing data, QIIME2 (2019.4) was firstly used for data optimization to remove the low-quality sequences with lengths shorter than 200 bp, average masses less than 25, and ambiguous bases. Using the dada2 method, only dereplication was applied to the high-quality sequences, which was equivalent to clustering with 100% similarities, and the OTU sequence with one and only one in all samples was removed. Each de-weighted sequence generated after quality control is called an ASV (corresponding to the OTU representing sequence), and the abundance table of these sequences in the sample is called the feature table (corresponding to the OTU table) [20]. Considering the differences in sequencing depth between different samples, the sequencing results were flattened according to the minimum sequencing volume in order to optimize the comparison between samples. Subsequently, species annotation and subsequent analysis of prokaryotic microorganisms and fungi were conducted based on the Green genes database [21].

## 3. Results and Discussion

### 3.1. Changes in α Diversity of Bacterial Communities 

In order to understand the changes of bacterial communities in coastal tidal wetlands after an *S. alterniflora* invasion, the bacterial composition and diversity were analyzed using classified sequences of all samples. Operational taxonomic units (OTUs) were denoised using QIIME2 DADA2 [22] and clustered using Vsearch [23] with a 97% similarity cut-off. The RDP FrameBot is used to correct insertion and deletion errors in nucleic acid sequences. A total of 1,890,167 raw sequences were generated from seventeen soil samples from three regions (eight collected from bare flats, four collected from areas of Sueada, five collected from areas invaded by *S. alterniflora*), and 1,541,492 amplicon sequence variants (ASVs) were screened. Finally, 1,175,770 high-quality prokaryotic 16S r RNA sequences were obtained from 17 soil samples. The length for all samples ranged from 366 to 435. 

**Figure 2:**
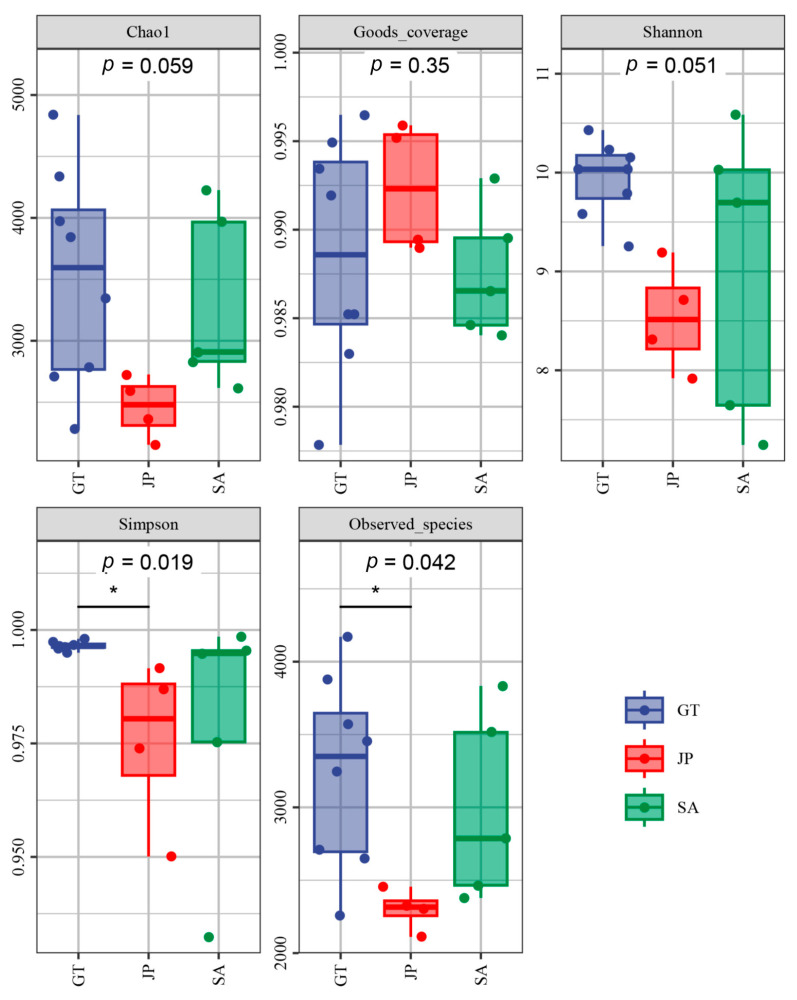
Five different types of indexes of soil samples form bare flats (GT), *Sueada salsa* areas (JP), and *S. alterniflora* Loisel areas (SA). The Chao1 and Observed_species indexes show community richness. The Shannon and Simpson indexes show the community diversity. Good’s coverage index shows the community coverage analysis of each sample. * indicates that there are significant differences between the two groups of data using dunn’s test.

The results of composition and diversity in the varied samples are shown in Figure 2, including the analysis of Chao1, Goods-coverage, Shannon, Simpson, and Observed_species. Community richness was represented by the Chao1 and Observed_species index. The Shannon and Simpson indexes were utilized to analyze the community diversity. In addition, community coverage analysis was acquired using Good’s coverage index to evaluate the bacterial diversity of each sample [24]. It was demonstrated that the samples covered all three areas well (Good-coverage > 0.985). A correlation of *p* = 0.059 (Kruskal-Wallis test) was observed between the OTUs and Chao1; thus, Chao1 can roughly indicate the total number of species in a sample. In general, there are significant differences among the three ecological areas. It showed that bacterial richness in *Sueada salsa* areas was the least, which was consistent with Observed_species (*p* = 0.042). The abundance and diversity of soil bacteria in *S. alterniflora* invasion areas were higher than that of the native species (*Sueada salsa* area), but lower than that of bare flat areas.

### 3.2. Changes in Bacterial Community Composition

**Figure 3:**
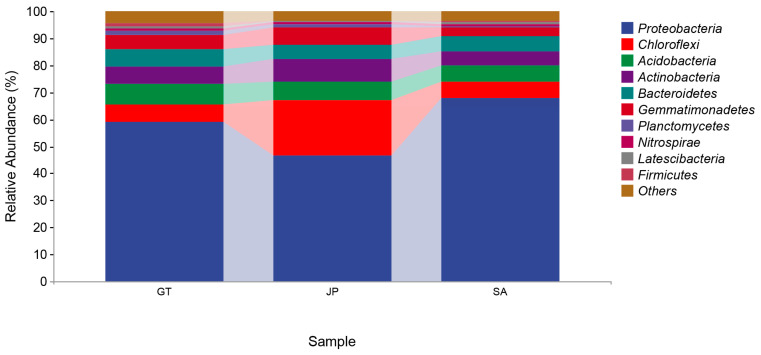
Taxonomic composition of soil bacterial communities at the phylum level in bare flats (GT), *Sueada salsa* areas (JP), and *S. alterniflora* Loisel areas (SA).

In terms of the composition of the soil bacterial community, 42 bacteria phyla were detected in 17 soil samples (Figure 3). The abundant phyla varied across all the samples, but generally included *Proteobacteria*, *Chloroflexi*, *Acidobacteria*, *Actinobacteria*, *Bacteroidetes,* and *Gemmatimonadetes*. These bacteria were made up, respectively, of 57.8%, 10.9%, 6.9%, 6.7%, 5.8%, and 5.1% of the soil bacterial community on average, which in total made up more than 90% of the bacterial community. Therein, *Proteobacteria* accounted for the highest proportion. Especially in the area invaded by *S. alterniflora*, the proportion of *Proteobacteria* was nearly 70%, which was much higher than that of two native ecosystems in coastal wetlands.

Nowadays, there is no comprehensive conclusion on the decomposition rate of organic carbon by microbiome [19,25]. However, previous studies on the assessment of bacterial carbon decomposition potential from the perspective of functional genes have shown that *Proteobacteria*, *Actinobacteria*, *Green Bayobacteria*, and *Bacteroidetes* are main bacterial groups involved in soil carbon metabolism in coastal wetland soil at the phylum level, among which *Proteobacteria* and *Actinobacteria* are the main contributors [26,27]. They had a strong carbon decomposition ability, which had the ability to degrade disaccharides, polysaccharides, and cellulose. In our study, it was clear that the *Proteobacteria* abundance in the *S. alterniflora* invasion area was close to the bare flat, and higher than that in the native *Sueada salsa* area (Figure 3). In addition, *Proteobacteria* occupied nearly 70% in the *S. alterniflora* invasion area (Figure 3). This meant that bacterial carbon metabolism activity was more active than that in the native areas, especially in the *Sueada salsa* area. As for *Actinobacteria*, the proportions of *Actinobacteria* in the three areas were all close and in the range of 5–8% (Figure 3). To sum up, when there are *S. alterniflora* invasions, the proportion of *Proteobacteria* in bare flat areas increased. Moreover, the total number of bacteria and the proportion of *Proteobacteria* in *Sueada salsa* areas were both increased according to Figure 2 and Figure 3. The changes in the bacterial community may enhance soil carbon decomposition. 

### 3.3. Similarities and Differences among Soil Bacterial Communities

To further demonstrate the effects of an *S. alterniflora* invasion on a soil bacterial community in a coastal tidal flat, the distribution characteristics of samples in three areas were visualized using NMDS. The Anosim test was used to determine the inter-group differences among bare flats, *Sueada salsa* areas, and *S. alterniflora* invasion areas [28,29]. The distance algorithm was performed with Jaccard.

**Figure 4:**
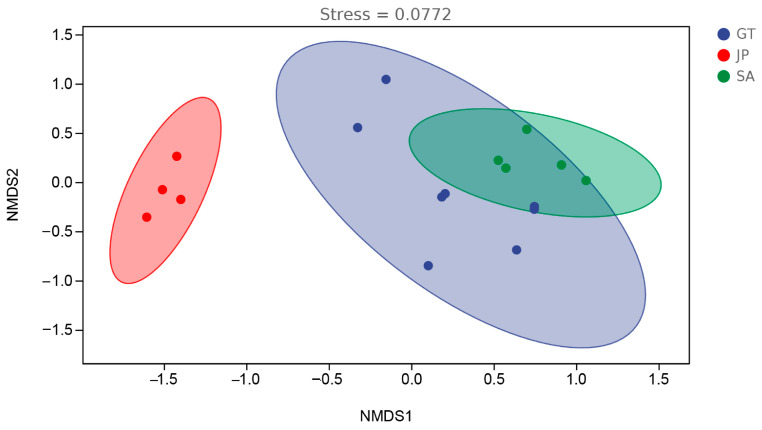
Visualization of the distribution characteristics of samples from bare flats (GT), *Sueada salsa* areas (JP), and *S. alterniflora* Loisel areas (SA) (confidence interval ≥ 0.95).

According to Figure 4 and Table 2, the stress value of NMDS was 0.0772, much less than 0.2, which means the NMDS result is highly reliable [30]. The soil bacterial community in the *Sueada salsa* area was significantly different from that in the other two areas, in particular a very low similarity to *S. alterniflora* (R_JP-SA_ = 1). Interestingly, the bare flats and *Sueada salsa* areas were both native ecological areas of the coastal wetland but showed a great difference in soil bacterial communities. The results revealed that the similarity of soil bacterial communities between bare flats and *Sueada salsa* areas (R_GT-JP_ = 0.914) was lower than that between bare flats and *S. alterniflora* invasion areas (R_GT-SA_ = 0.278). A more similar soil microbiome composition would be more conducive to plant growth. This may also be one of the reasons why *S. alterniflora* has been able to bio-invasively replace the ecological niche of proto-species such as *Sueada salsa*.

### 3.4. Verification of Similarities and Differences of Soil Bacterial Communities

**Figure 5:**
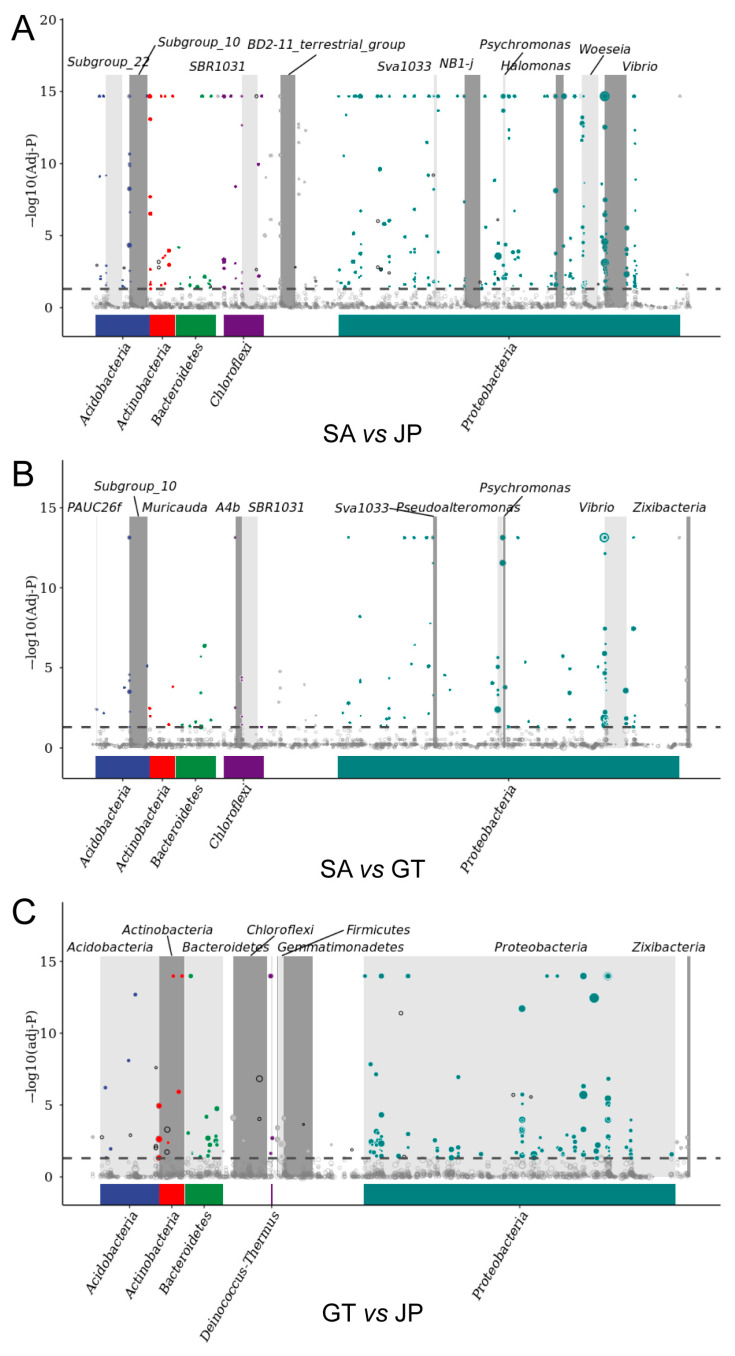
MetagenomeSeq analysis looked for significant differences in species composition between bare flats (GT), *Sueada salsa* areas (JP), and *S. alterniflora* Loisel areas (SA). (**A**) Differences in gene expression in bacterial communities between SA and JP. (**B**) Differences in gene expression in bacterial communities between SA and GT. (**C**) Differences in gene expression in bacterial communities between GT and JP. The top icon shows the most distinct phylum. The color of the points represents the phylum to which it belongs, which is consistent with the color of the phylum below it.

Three groups (GT, JP, SA) of undrawn ASV data were divided into two groups and imported into zero-inflated log-normal models using the fitFeature Model function, respectively. The logarithm of genetic difference (−1 < log_2_FC < 1) was calculated, and the results were obtained (Figure 5). Among them, the round dot represented the abundance of the genus, which above the dotted line represented having significant differences in this genus. Through this analysis, the differences between the soil bacterial community in the *S. alterniflora* invasion area and the wetland native area, at the bacterial genus level, were found. Then, the differences were clustered to verify the results of the differences at the bacterial phylum level. Each of these points represents a different bacterial genus. The larger the point, the more significant the difference in the bacterial genus between the two groups. 

It can be clearly seen that, although the genus was different, the differences in *Proteobacteria* were the most significant in the whole bacterial community. The *S. alterniflora* invasion area had the highest abundance. The lowest abundance was found in the *Sueada salsa* area. There were very significant differences between the *S. alterniflora* invasion area and the *Sueada salsa* area. In addition, it can also be roughly seen that the difference between the *S. alterniflora* invasion area and the bare flat is smaller than that between the bare flat and the *Sueada salsa* area, which is also consistent with the previous conclusion—the *S. alterniflora* invasion area and the bare flat had high similarities.

**Figure 6:**
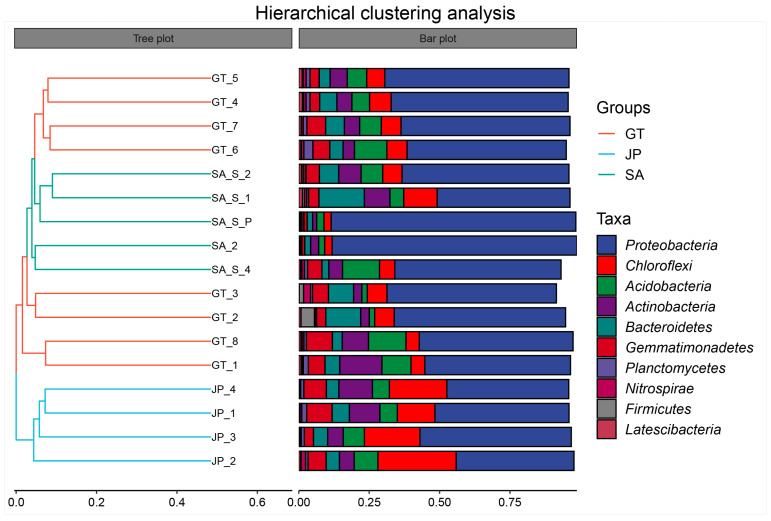
The hierarchical clustering analysis of bare flats (GT), *Sueada salsa* areas (JP), and *S. alterniflora* Loisel areas (SA). On the left is the hierarchical clustering tree diagram, where samples are clustered according to their similarity. The shorter the branch length between samples, the more similar the two samples are. On the right is a stacked bar chart of the top 10 genera in abundance.

To further determine the similarity between soil communities in three areas, a hierarchical clustering analysis was performed for all samples. According to Figure 6, samples from the *Sueada salsa* area were significantly removed from the other two areas. It was also proved that although bare flat sand *Sueada salsa* areas were both the native ecological areas of the coastal wetland, the soil bacterial community composition of bare flats is more similar to that of *S. alterniflora* invasion areas. Especially, the intra-group sample distances between some bare flat samples were higher than the distances between the bare flat and *S. alterniflora* invasion area samples. This may be that bacterial communities in bare flats were significantly affected by factors such as tides, rivers, distance from the coast, and so on [31,32]. So, the soil bacterial community composition of some bare flats has been suitable for the long-term growth of *S. alterniflora*.

### 3.5. Possible Relationship between Soil Carbon Content Changes and Bacterial Community Changes

To evaluate changes in soil carbon storage induced by an *S. alterniflora* invasion, the location information, pH, EC, and carbon content (TC, IC, and SOC) of the samples were detected and are shown in Table 1. Since all the areas were located near the same dike, the tidal cover, tidal water depth, and hydrological characteristics in all three ecological areas were very similar. The results revealed that the soil in the three areas was weak in alkaline. The pH in the area invaded by *S. alterniflora* was the lowest, which was consistent with the conclusion of previous studies that soil acidification was caused by *S. alterniflora* invasions [33]. Results of SOC suggested that the *Sueada salsa* area had the lowest concentration, and the bare flat was next. Relative to the *Sueada salsa* area and the bare flat, it showed the highest concentration invaded by *S. alterniflora*. These observations revealed that the soil of *S. alterniflora* invasion areas can store more organic carbon. Moreover, the soil bacterial abundance was lower in *S. alterniflora* invasion areas than in bare flats (Figure 2), suggesting that *S. alterniflora* invasion areas were more suitable for soil organic carbon storage than bare flats. Notably, according to Figure 3, *Proteobacteria* occupied a high proportion in the soil of the *S. alterniflora* invasion area. *Proteobacteria* had strong organic carbon decomposition ability, which usually reduces organic carbon content. Nevertheless, the high SOC content in the soil of *S. alterniflora* invasion areas may be related to the properties of *S. alterniflora* and the characteristics of the bacterial communities in coastal wetlands, which is not only associated with one factor. For one thing, previous studies have provided evidence that *S. alterniflora* had a stronger carbon fixation capacity than other wetland plants and was decomposed more slowly [34,35], which provided sufficient organic matter for bacteria. It partly explained the reason why soil in *S. alterniflora* invasion areas had higher SOC and was consistent with SOC contents in our study. For another, higher bacterial carbon metabolism activity did not lead to lower SOC in our study. This was possibly related to the decomposition ability of bacterial communities in coastal wetlands which had a lower ability to break down certain substances, such as monosaccharides, carboxylic acids, alcohols, and phenols [36,37,38]. Organic carbon is well stored in these forms.

However, in terms of TC, the soil carbon content in *S. alterniflora* invasion areas was lower than that in *Sueada salsa* areas. This was because *Sueada salsa* areas’ soil contained higher IC. The *S. alterniflora* invasion could cause soil acidification, which is not conducive to the storage of inorganic carbon. That is to say, the soil in *Sueada salsa* areas can store more carbon, especially a large amount of inorganic carbon. Compared with *S. alterniflora* invasion areas, the bacterial richness in *Sueada salsa* areas was lower (Figure 2). These were both more conducive to soil carbon storage performance.

To sum up, although an *S. alterniflora* invasion could bring some benefits to the bare flats, the carbon storage capacity of *S. alterniflora* soil was weaker than that of *Sueada salsa* area soil. When *S. alterniflora* flourished in coastal wetlands and threatened the survival of other species such as *Sueada salsa*, it reduced the carbon storage capacity of coastal wetlands.

The changes of SOC caused by an *S. alterniflora* invasion is directly related to the changes of soil bacterial communities mentioned above. In coastal wetlands, *Proteobacteria* and *Actinobacteria* were the main bacterial groups involved in soil carbon decomposition, which could secrete a variety of organic carbon-degrading enzymes [39]. The *S. alterniflora* invasion led to the increase in SOC in coastal wetlands, which would lead to the increase in *Proteobacteria* richness and its proportion in the soil bacterial community. As for why *S. alterniflora* invasion areas had higher SOC when the bacterial community had higher carbon decomposition capacity, this may be related to the weak ability of soil bacteria to decompose monosaccharides, carboxylic acids, alcohols, phenols, and other substances in coastal wetlands [36,37,38]. A large amount of organic carbon may be stored in these forms.

## 4. Conclusions

In this paper, one of the other reasons why *S. alterniflora* easily invades coastal wetlands and the changes in bacterial community affected in soil carbon storage were discussed. In detail, it has a high similarity between the naturally formed bare flat soil bacterial community, and in the soil environment where *S. alterniflora* grows, it does so for a long time. *S. alterniflora* is well adapted to the soil bacterial environment in bare flats.

The invasion and expansion of *S. alterniflora* would lead to changes in soil bacterial communities and soil carbon storage in coastal wetlands. One of the most significant was an increase in *Proteobacteria*. At the same time, *S. alterniflora* invasions increased soil organic carbon content in coastal wetlands and also provided sufficient organic matter for bacteria. The combined effects of the two may further improve soil carbon decomposition rates in coastal wetlands. Due to lack of ability to break down some particular matters, a lot of organic carbon may be stored in the form of monosaccharides, carboxylic acids, alcohols, phenols, etc.

In addition, the *S. alterniflora* invasion could also lead to soil acidification in coastal wetlands, thus reducing soil inorganic carbon content, which makes the carbon storage capacity of soil invaded by *S. alterniflora* lower. Thus, when *S. alterniflora* threatened the survival of other native species in coastal wetlands, it reduces the carbon storage capacity of coastal wetland.

## Figures and Tables

**Table 1:** Sample collection area information and partial physicochemical properties information.

Sample	GT	JP	SA
Vegetation	Bare flat	*Sueada* salsa	*Spartina alterniflora* Loisel
Location	32°45′ N, 120°57′ E	32°52′ N, 120°56′ E	32°47′ N, 120°57′ E
pH	8.34 ± 0.39	8.52 ± 0.05	7.68 ± 0.10
EC (mS/cm)	43.4 ± 2.7	34.8 ± 4.7	36.8 ± 2.7
TC (mg/g)	12.11 + 1.56	14.42 ± 1.53	13.45 ± 2.64
IC (mg/g)	2.17 ± 0.46	7.27 ± 1.02	3.16 ± 0.73
SOC (mg/g)	9.94 ± 0.23	7.15 ± 0.63	10.29 ± 0.35

**Table 2:** Anosim was used to verify difference between bare flats (GT), *Sueada salsa* areas (JP), and *S. alterniflora* Loisel areas (SA).

Group1	Group2	Sample Size	R	*p*-Value	q-Value
GT	JP	12	0.914	0.004	0.011
GT	SA	13	0.278	0.034	0.034
JP	SA	9	1	0.007	0.011

## Data Availability

Data is unavailable due to privacy or ethical restrictions.

## References

[1] Finlayson C.M. (2010). Coastal wetlands and climate change: The role of governance and science. Aquat. Conserv..

[2] Liu M., Mao D., Wang Z., Li L., Man W., Jia M., Ren C., Zhang Y. (2018). Rapid Invasion of *Spartina alterniflora* in the Coastal Zone of Mainland China: New Observations from Landsat OLI Images. Remote Sens..

[3] Mcleod E., Chmura G.L., Bouillon S., Salm R., BjöRk M., Duarte C.M., Lovelock C.E., Schlesinger W.H., Silliman B.R. (2011). A blueprint for blue carbon: Toward an improved understanding of the role of vegetated coastal habitats in sequestering CO2. Front. Ecol. Environ..

[4] Bastviken D., Tranvik L.J., Downing J.A., Crill P.M., Enrich-Prast A. (2011). Freshwater methane emissions offset the continental carbon sink. Science.

[5] Macreadie P.I., Nielsen D.A., Kelleway J.J., Atwood T.B., Seymour J.R., Petrou K., Connolly R.M., Thomson A.C.G., Trevathan-Tackett S.M., Ralph P.J. (2017). Can we manage coastal ecosystems to sequester more blue carbon. Front. Ecol. Environ..

[6] Zhou H.X., Liu J.E., Qin P. (2009). Impacts of an alien species (*Spartina alterniflora*) on the macrobenthos community of Jiangsu coastal inter-tidal ecosystem. Ecol. Eng..

[7] Chung C.H., Zhuo R.Z., Xu G.W. (2004). Creation of *Spartina* plantations for reclaiming Dongtai, China, tidal flats and offshore sands. Ecol. Eng..

[8] Yang R.M. (2020). Characterization of the salt marsh soils and visible-near-infrared spectroscopy along a chronosequence of *Spartina alterniflora* invasion in a coastal wetland of eastern China. Geoderma.

[9] Li B., Liao C., Zhang X., Chen H., Wang Q., Chen Z., Gan X., Wu J., Zhao B., Ma Z. (2009). *Spartina alterniflora* invasions in the Yangtze River estuary, China: An overview of current status and ecosystem effects. Ecol. Eng..

[10] Meng W., Feagin R.A., Innocenti R.A., Hu B., Li H. (2020). Invasion and ecological effects of exotic smooth cordgrass *Spartina alterniflora* in China. Ecol. Eng..

[11] Wan S., Qin P., Liu J., Zhou H. (2009). The positive and negative effects of exotic *Spartina alterniflora* in China. Ecol. Eng..

[12] Qin P., Li S.Y. (2012). Two sides of *Spartina alterniflora* and its ecological control. J. Biosaf..

[13] Zhao Y., Wang S., Yang W., Li Y., Kong F. (2022). Research progress and prospects for the control of *Spartina alterniflora* in China. J. Biosaf..

[14] Levin L.A., Neira C., Grosholz E.D. (2006). Invasive Cordgrass Modifies Wetland Trophic Function. Ecology.

[15] Ma Z., Gan X., Cai Y., Chen J., Li B. (2011). Effects of exotic Spartina alterniflora on the habitat patch associations of breeding saltmarsh birds at Chongming Dongtan in the Yangtze River estuary, China. Biol. Invasions.

[16] Zhang C.B., Liu W.L., Luo B., Guan M., Wang J., Ge Y., Chang J. (2020). *Spartina alterniflora* invasion impacts denitrifying community diversity and functioning in marsh soils. Geoderma.

[17] Yu C., Cao J., Du W., Zhu Z., Xu M. (2022). Changes in the population and functional profile of bacteria and fungi in the rhizosphere of *Suaeda salsa* is driven by invasion of *Spartina alterniflora*. Ecol. Indic..

[18] Sundberg C., Al-Soud W.A., Larsson M., Alm E., Yekta S.S., Svensson B.H., Sorensen S.J., Karlsson A. (2013). 454 pyrosequencing analyses of bacterial and archaeal richness in 21 full-scale biogas digesters. FEMS Microbiol. Ecol..

[19] De Mares M.C., Sipkema D., Huang S.X., Bunk B., Overmann J., van Elsas J.D. (2017). Host Specificity for Bacterial, Archaeal and Fungal Communities Determined for High- and Low-Microbial Abundance Sponge Species in Two Genera. Front. Microbiol..

[20] Benjamini Y., Hochberg Y. (1995). Controlling the False Discovery Rate: A Practical and Powerful Approach to Multiple Testing. J. R. Stat. Soc. B.

[21] DeSantis T.Z., Hugenholtz P., Larsen N., Rojas M., Brodie E.L., Keller K., Huber T., Dalevi D., Hu P., Andersen G.L. (2006). Greengenes, a chimera-checked 16S rRNA gene database and workbench compatible with ARB. Appl. Environ. Microbiol..

[22] Callahan B.J., McMurdie P.J., Rosen M.J., Han A.W., Johnson A.J.A., Holmes S.P. (2016). DADA2: High-resolution sample inference from Illumina amplicon data. Nat. Methods.

[23] Rognes T., Flouri T., Nichols B., Quince C., Mahe F. (2016). VSEARCH: A versatile open source tool for metagenomics. PeerJ.

[24] Zhang S., Pang S., Wang P., Wang C., Guo C., Addo F.G., Li Y. (2016). Responses of bacterial community structure and denitrifying bacteria in biofilm to submerged macrophytes and nitrate. Sci. Rep..

[25] Liang C., Schimel J.P., Jastrow J.D. (2017). The importance of anabolism in microbial control over soil carbon storage. Nat. Microbiol..

[26] Kang H., Kim S.Y., Freeman C., DeLaune R.D., Reddy K.R., Richardson C.J., Megonigal J.P. (2013). Enzyme Activities Methods in Biogeochemistry of Wetlands. Madison: Soil Science Society of America.

[27] Zhang Z., Han P., Zheng Y., Jiao S., Dong H., Liang X., Gao D., Niu Y., Yin G., Liu M. (2022). Spatiotemporal Dynamics of Bacterial Taxonomic and Functional Profiles in Estuarine Intertidal Soils of China Coastal Zone. Microb. Ecol..

[28] Clarke K.R. (1993). Non-parametric multivariate analyses of changes in community structure. Aust. J. Ecol..

[29] Warton D.I., Wright S.T., Wang Y. (2012). Distance-based multivariate analyses confound location and dispersion effects. Methods Ecol. Evol..

[30] Legendre P., Gallagher E.D. (2001). Ecologically meaningful transformations for ordination of species data. Oecologia.

[31] Bernal B., Mitsch W.J. (2012). Comparing carbon sequestration in temperate freshwater wetland communities. Glob. Chang. Biol..

[32] Bernal B., Mitsch W.J. (2013). Carbon sequestration in freshwater wetlands in Costa Rica and Botswana. Biogeochemistry.

[33] Zhang G., Bai J., Zhao Q., Jia J., Wang X. (2021). Soil carbon storage and carbon sources under different *Spartina alterniflora* invasion periods in a salt marsh ecosystem. Catena.

[34] Liao C., Luo Y., Jiang L., Zhou X., Wu X., Fang C., Chen J., Li B. (2007). Invasion of *Spartina alterniflora* Enhanced Ecosystem Carbon and Nitrogen Stocks in the Yangtze Estuary, China. Ecosystems.

[35] Liao C.Z., Luo Y.Q., Fang C.M., Chen J.K., Li B. (2008). Litter pool sizes, decomposition, and nitrogen dynamics in *Spartina alterniflora*-invaded and native coastal marshlands of the Yangtze Estuary. Oecologia.

[36] Yang W., Jeelani N., Leng X., Cheng X., An S. (2016). *Spartina alterniflora* invasion alters soil microbial community composition and microbial respiration following invasion chronosequence in a coastal wetland of China. Sci. Rep..

[37] Pei L., Ye S., Yuan H., Pei S., Xie S., Wang J. (2020). Glomalin-related soil protein distributions in the wetlands of the Liaohe Delta, Northeast China: Implications for carbon sequestration and mineral weathering of coastal wetlands. Limnol. Oceanogr..

[38] Neori A., Agami M. (2017). The Functioning of Rhizosphere Biota in Wetlands—A Review. Wetlands.

[39] Yangyao J., Chen H., Wang Y., Kan P., Yao J., Zhang D., Sun W., Yao Z. (2023). Metagenomic insights into the functional genes across transects in a typical estuarine marsh. Sci. Total Environ..

